# Urinary Neutrophil Gelatinase-associated Lipocalin in the evaluation of Patent Ductus Arteriosus and AKI in Very Preterm Neonates: a cohort study

**DOI:** 10.1186/s12887-016-0761-0

**Published:** 2017-01-10

**Authors:** Anna Sellmer, Bodil H. Bech, Jesper V. Bjerre, Michael R. Schmidt, Vibeke E. Hjortdal, Gitte Esberg, Søren Rittig, Tine B. Henriksen

**Affiliations:** 1 Department of Pediatrics, Aarhus University Hospital, Palle Juul-Jensens Boulevard 99, DK 8200 Aarhus, Denmark; 2 Perinatal Epidemiology Research Unit, Department of Pediatrics, Aarhus University Hospital, Palle Juul-Jensens Boulevard 99, DK 8200 Aarhus, Denmark; 3 Department of Public Health, Section for Epidemiology, Aarhus University, Bartholins Allé 2, DK-8000 Aarhus, Denmark; 4 Department of Cardiology, Aarhus University Hospital, Palle Juul-Jensens Boulevard 99, DK 8200 Aarhus, Denmark; 5 Department of Cardiothoracic surgery, Aarhus University Hospital, Palle Juul-Jensens Boulevard 99, DK 8200 Aarhus, Denmark

**Keywords:** Acute kidney injury, Neutrophil gelatinase-associated lipocalin, Patent ductus arteriosus, Very preterm neonates

## Abstract

**Background:**

A patent ductus arteriosus (PDA) is frequently found in very preterm neonates and is associated with increased risk of morbidity and mortality. A shunt across a PDA can result in an unfavorable distribution of the cardiac output and may in turn result in poor renal perfusion. Urinary Neutrophil Gelatinase-associated Lipocalin (U-NGAL) is a marker of renal ischemia and may add to the evaluation of PDA. Our primary aim was to investigate if U-NGAL is associated with PDA in very preterm neonates. Secondary, to investigate whether U-NGAL and PDA are associated with AKI and renal dysfunction evaluated by fractional excretion of sodium (FENa) and urine albumin in a cohort of very preterm neonates.

**Methods:**

A cohort of 146 neonates born at a gestational age less than 32 weeks were **c**onsecutively examined with echocardiography for PDA and serum sodium, and urine albumin and sodium were measured on postnatal day 3 and U-NGAL and serum creatinine day 3 and 6. AKI was defined according to modified neonatal Acute Kidney Injury Network (AKIN) criteria. The association between U-NGAL and PDA was investigated. And secondly we investigated if PDA and U-NGAL was associated with AKI and renal dysfunction.

**Results:**

U-NGAL was not associated with a PDA day 3 when adjusted for gestational age and gender. A PDA day 3 was not associated with AKI when adjusted for gestational age and gender; however, it was associated with urine albumin. U-NGAL was not associated with AKI, but was found to be associated with urine albumin and FENa.

**Conclusions:**

Based on our study U-NGAL is not considered useful as a diagnostic marker to identify very preterm neonates with a PDA causing hemodynamic changes resulting in early renal morbidity. The interpretation of NGAL in preterm neonates remains to be fully elucidated.

## Background

A patent ductus arteriosus (PDA) in preterm neonates is associated with an increased risk of morbidity and mortality [1, 2]. On day 3 of life, up to 50% of neonates born before 32 weeks of gestation have a PDA [1, 3]. However, not every PDA challenges the preterm neonate. We have previously demonstrated that a PDA diameter above 1.5 mm on postnatal day 3 can be used to identify a clinically significant PDA [1]. However, further diagnostic tools are needed in order to understand the potential impact of PDA presence and optimize handling of PDA in very preterm neonates.

Neutrophil gelatinase-associated lipocalin (NGAL) is expressed in low concentrations in many organs and the expression is up-regulated during infection, inflammation, and ischemia [4, 5]. Furthermore, NGAL is believed to be involved in renal development [6]. Urinary NGAL is produced in the renal tubule of the thick ascending limb of Henle and the collecting ducts [7]. In preterm neonates, NGAL has been suggested to be associated with Acute Kidney Injury (AKI) [8, 9].

U-NGAL has been hypothesized to be increased in the presence of a PDA [10]. A shunt across a PDA may result in an unfavorable distribution of the cardiac output between the pulmonary and the systemic circulation. This may in turn result in poor renal perfusion. This could cause AKI or renal dysfunction due to ischemia which could increase U-NGAL [11].

The aim of this study was to evaluate if U-NGAL can be used as a diagnostic marker to identity very preterm neonates with a PDA causing hemodynamic changes resulting in renal morbidity within the first week of life. To do this we investigated if U-NGAL is associated with PDA in very preterm neonates. Secondly we investigated whether U-NGAL and PDA were associated with AKI and renal dysfunction evaluated by fractional excretion of sodium and urine albumin in a cohort of very preterm neonates.

## Methods

### Study population

**Fig. 1:**
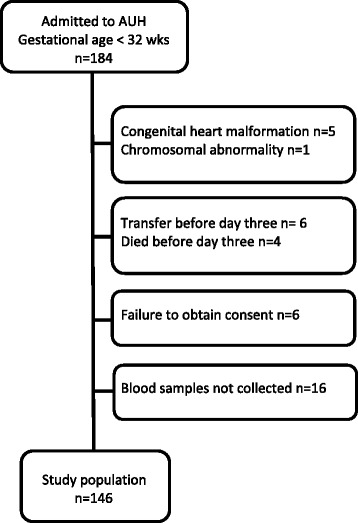
Study population from 184 very preterm neonates admitted to Aarhus University Hospital (AUH)

The study population has previously been described [1], in brief, newborns born at gestational age below 32 weeks admitted to Aarhus University Hospital were eligible. We excluded neonates with chromosomal abnormalities or congenital heart malformations other than atrial septum defects from the study (Fig. 1).

### Data collection and definitions

Irrespective of clinical symptoms, all very preterm neonates had an echocardiography on day 3 (equal to 72 h). Urine samples and blood samples were collected day 3 and 6. Urine was collected with a cotton ball placed at the perineum [8, 9, 12]. The diaper was checked at least every 4 h and the cotton transferred to a refrigerator. Cotton was centrifuged at 1000 rpm for 1 min and the urine was stored at −80 °C within 24 h after collection.

From the patient medical records we collected the following clinical details: gestational age at birth (determined by ultrasound), birth weight, multiple births, Apgar score, early onset sepsis (defined as seven days of antibiotics initiated within the first 3 days after birth) [13, 14], surfactant administration, packed red blood cell transfusion (within the first 3 days), use of inotropes (within the first 3 days), maternal preeclampsia, antenatal steroid administration, and mode of delivery.

### Clinical guidelines

Management of the PDA was in accordance with institutional clinical guideline. Treatment with Ibuprofen (Pedea, Orphan Europe SARL Puteaux, France) was initiated based on detection by echocardiography alone if the gestational age at birth was less than 28 weeks; otherwise treatment was initiated based on a combination of specific echocardiographic findings and clinical symptoms. Ibuprofen was never administered before day 3.

### Echocardiographic measurements

A complete diagnostic echocardiography was performed by two senior pediatric echocardiographers (J Bjerre and MR Schmidt) by use of standard neonatal windows. A Philips IE33 Ultrasound machine with a 12-MHz cardiology probe (Philips Healthcare, Andover, Massachusetts, USA) was used. A PDA was defined as present if flow could be visualized on colour Doppler. The PDA diameter was measured in two-dimensional mode at the most narrow point. A mean value of three cardiac cycles was calculated. A large PDA was defined as a PDA with a diameter of 1.5 mm or more and a small PDA as a PDA with a diameter below 1.5 mm.

### U-NGAL measurement

The U-NGAL test Reagent Kit from BioPorto was used (BioPorto, Copenhagen, Denmark) as routine analysis on the Cobas 6000 analyzer system (Roche, Basel, Switzerland) at the Department of Biochemistry, Aarhus University Hospital. Also, serum creatinine (SCr), serum sodium, urinary albumin and sodium were measured as routine analyses at the Department. AKI was defined according to the definition proposed by Jetton et Askenazi based on the neonatal acute kidney injury (AKIN) classification [15–17]. AKI Stage 0: no change or rise SCr < 0.3 mg/dl; Stage 1: increase SCr 0.3 mg/dl or increase SCr 150–200% from previous trough value; Stage2: increase SCr 200–300% from previous trough value; Stage 3: increase SCr 200% from previous trough value or 2.5 mg/dl or recipient of dialysis [15]. Six stable neonates only had one SCr measured, this was within normal range and they were categorized as not having AKI. To evaluate renal dysfunction we measured urine albumin and calculated fractional excretion of sodium (FENa). Fractional excretion of sodium was calculated using the following formula: FENa (%) = (UNa/ PNa) * (SCr/ UCr) * 100, where UNa = urine sodium (mmol/l); PNa = plasma sodium (mmol/l); SCr serum creatine (μmol/l); and UCr = urine creatinine (μmol/l).

### Statistical analysis

U-NGAL, urine albumin, SCr, and FENa were transformed by the natural logarithm (ln) to normalize the distribution of residuals. Geometric means were compared using the Student’s t-test and proportions using Fisher’s exact test. Univariate and multivariate log-linear regression was performed in order to examine the association between U-NGAL and PDA day 3, PDA size, perinatal characteristics, and sepsis. We consequently exponentiated the differences estimated on the natural log scale to obtain ratios of the medians. The formula (exp^beta^ -1) * 100% was used to express the percent change. These ratios are presented as crude and adjusted percent change per unit of explanatory variable or difference from reference group with 95% confidence interval (CI). Univariate and multivariate log-log regression was performed to examine the association between U-NGAL and parameters of renal dysfunction. The formula (1.01^beta^-1)* 100% was used to express the percent change in U-NGAL per 1% change in urine albumin, SCr or FENa with 95% CI. Univariate and multivariate logistic regression was performed to examine the association between AKI and PDA. Odds ratio (OR) was presented with 95% CI. Estimates were adjusted for gestational age as a continuous variable (weeks) and gender and sepsis as dichotomized variables. Robust cluster standard errors were used in order to take into account the correlation between twins. Data were analyzed with STATA special edition version 11 (College Station, Texas, USA). All tests were two-sided. *P*-values of less than 0.05 were considered statistically significant.

## Results

### Patient characteristics

A total of 146 neonates were included in the study (Fig. 1). A PDA day 3 was found in 67 (46%) neonates on day 3 and 41 (61%) of neonates with PDA had a large PDA. Characteristics of the 146 very preterm neonates in the study are found in Table 1. Neonates with large and small PDA had comparable characteristics (data not shown).

**Table 1:** Characteristics of 146 very preterm neonates postnatal day 3

	no PDA	PDA	*P* value
	(*n* = 79)	(*n* = 67)	
Gestational age weeks, median (range)	29 (24; 31)	27 (23; 31)	<0.01
Birth weight grams, median (range)	1190 (470;2160)	940 (570;1840)	<0.05
Singleton, number (%)	59 (75)	32 (48)	<0.01
SGA, number (%)	28 (35)	14 (21)	0.07
Sex (female:male)	27:52	28:39	0.39
Apgar score at 1 min, median (IQR)	8 (5;10)	7 (5;8)	<0.05
Apgar score at 5 min, median (IQR)	9 (7;10)	10 (9;10)	0.30
Sepsis, number (%)	9 (11)	13 (20)	0.25
Surfactant, number (%)	29 (37)	37 (55)	<0.05
PRBC transfusion, number (%)	8 (10)	16 (24)	<0.05
Inotropes, number (%)	5 (6)	7 (11)	0.39
Preeclampsia, number (%)	27 (34)	7 (11)	<0.01
Antenatal steroids, number (%)	75 (97)	61 (95)	0.66
Cesarean delivery, number (%)	56 (71)	44 (66)	0.59

*SGA* small-for-gestational-age (birth weight below 3rd percentile for gestational age); Sepsis defined as 7 days of antibiotics initiated before day 3; *PRBC* packed red blood cell transfusion within first 3 days of life; Inotropes used within the first 3 days of life

### U-NGAL in neonates with PDA

**Fig. 2:**
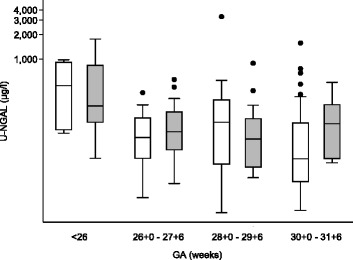
Urine neutrophil gelatinase-associated lipocalin (U-NGAL) in 146 very preterm neonates with no PDA (*white*) and PDA (*grey*) assessed at postnatal day 3

U-NGAL levels day 3 in neonates with PDA and no PDA on day 3 in four gestational age groups are found in Fig. 2. The highest level of U-NGAL day 3 was found in neonates with a small PDA compared to neonates with no PDA or a large PDA day 3 (Table 2). Median U-NGAL day 3 was 53% higher in neonates with a PDA compared to neonates without a PDA (Table 3). However, after adjusting for gender, gestational age, and sepsis by multivariate linear regression the association was no longer present. And no association was found between PDA or PDA size day 3 and U-NGAL.

**Table 2:** U-NGAL, SCr, FENa, urine albumin, and AKI in 146 very preterm neonates postnatal day 3

	no PDA	PDA	small PDA	large PDA
	*n* = 79	*n* = 67	*n* = 26	*n* = 41
U-NGAL day 3 (μg/l)	104 (79; 136)	158 (125; 201)*	177 (117; 266)*	148 (110; 200)*
U-NGAL day 6 (μg/l)	87 (63; 118)	139 (103; 190)*	166 (103; 267)*	126 (83; 190)
SCr (μmol/l)	78 (75; 82)	78 (75; 82)	78 (73; 84)	78 (74; 83)
Urinary albumin (mg/l)	29 (24; 36)	62 (51; 77)*	56 (41; 76)*	67 (51; 90)*
FENa (%)	2.6 (2.3; 2.9)	3.6 (3.2; 4.2)*	3.8 (3.0; 4.8)*	3.6 (3.0; 4.2)*
AKI, number (%)	1 (1)	6 (9)*	2 (8)	4 (10)*

*NGAL* urine neutrophil gelatinase-associated lipocalin, *SCr* serum creatinine, *AKI* acute kidney injury, *FENa* fractional excretion of sodium, *PDA diameter <1.5 mm* small PDA, *PDA diameter ≥1.5 mm* large PDA. Values are geometric mean (95% confidence interval) or number (percentage). *p < 0.05 compared to neonates with no PDA. U-NGAL day 6 was only measured in 126 neonates

**Table 3:** Influence of perinatal characteristics, sepsis, renal parameters, AKI, and PDA on U-NGAL in 146 very preterm neonates

		Percent change in U-NGAL Day 3	
	Crude	Adjusted GA, gender	Adjusted GA, gender, sepsis
Per gestational week	−17 (−23; −10)		
Girls only	−12 (−22; −0.2)		
Boys only	−20 (−27; −12)		
Male gender vs. female	−58 (−70; −40)		
Sepsis vs. no sepsis	329	240 (103; 466)	
Per percent increase SCr	0.4	0.3 (−0.6; 1.2)	0.7 (−0.2; 1.5)
Per percent increase urine albumin	0.6	0.5 (0.3; 0.7)	0.5 (0.3; 0.6)
Per percent increase FENa	0.7	0.5 (0.2; 0.8)	0.3 (0.0; 0.6)
PDA vs. no PDA day 3	53	0.3 (−30; 40)	0.4 (−27; 35)
Small PDA vs. no PDA	70	14 (−27; 76)	0.0 (−32; 47)
Large PDA vs. no PDA	42	−5 (−34; 38)	2.3 (−32; 41)
AKI vs. no AKI	−15	−18 (−63; 80)	−25 (−63; 52)
AKI vs. no AKI	34^a^	24 (−60; 295)	19 (−60; 269)

Percent change in U-NGAL (95% CI) concentration by one unit increase in gestational age, by 1% increase in SCr, urinary albumin, and FENa and difference in concentration between groups (male vs. female, sepsis vs. no sepsis, PDA vs. no PDA, AKI vs. no AKI). ^a^U-NGAL measured day 6 in 126 neonates all other U-NGAL was measured day 3

*U-NGAL* urine neutrophil gelatinase-associated lipocalin, *GA* gestational age, *SCr* serum creatinine, *FENa* fractional excretion of sodium, *AKI* acute kidney injury, *PDA diameter < 1.5 mm* small PDA, *PDA diameter ≥ 1.5 mm* large PDA

### AKI in neonates with PDA

A total of seven neonates were found to have AKI. AKI was found more frequently in neonates with PDA compared to neonates with no PDA and especially in neonates with a large PDA compared to no PDA day 3 (Table 2). However, when adjusted for gestational age no association between PDA day 3 and AKI was found. The odds of AKI decreased some 40% for every 1 week increase in gestational age at birth (OR = 0.64; 95% CI 0.4–0.96).

Mean SCr, urinary albumin, and FENa are described in Table 2. No difference in SCr was found between PDA groups. We found that urine albumin and FENa was higher in neonates with PDA day 3 compared to neonates with no PDA. On regression we found PDA and PDA size to be associated with urine albumin also when adjusted for adjusting for gender, gestational age, and sepsis. No association between PDA or PDA size and FENa was found when adjusted for gestational age.

### U-NGAL in neonates with AKI

We found no association between U-NGAL measured day 3 and AKI (Table 3). In 126 neonates we measured U-NGAL day 6. However, also on day 6 we found no association between U-NGAL and AKI. No association between U-NAGL day 3 and SCr was found. We did find an association between U-NGAL day 3 and both urine albumin and FENa also when adjusting for gender, gestational age, and sepsis.

## Discussion

In 146 consecutively examined very preterm neonates, we found that the mere presence of a PDA and the PDA size day 3 was not associated with U-NGAL after adjusting for gestational age and gender. AKI was found more often in neonates with PDA, but after adjusting for gestational age there was no association between PDA day 3 and AKI. We found that neonates with PDA day 3 had higher urine albumin compared to neonates with no PDA. PDA and PDA size was associated with urine albumin also when adjusted for gestational age, gender, and sepsis. U-NGAL was not associated with AKI, it was however found to be associated with both urine albumin and FENa.

Tosse et al. found that U-NGAL in neonates with a birth weight below 1,500 g was higher if a PDA that needed either medical or surgical intervention was present than in neonates with no PDA or a PDA with no need for intervention [10]. They diagnosed the PDA by echocardiography on day 1–3 but the need for medical or surgical intervention was based on respiratory setback, oliguria or failure to thrive. Recently PDA diameter has been recognized as an important marker to evaluate the magnitude and clinical significance of the PDA [1, 18–20]. We used a PDA diameter above 1.5 mm on day 3 to identify the clinically significant PDA as we have previously shown that it is associated with severe morbidity and mortality [1]. Markers of organ dysfunction prior to clinical morbidity are needed in order to evaluate the PDA. The hemodynamic effects of a PDA may cause renal hypoperfusion and renal ischemia leading to AKI [21, 22]. Also management of the PDA including Indomethacin, Ibuprofen or fluid restrictions may alter renal function or cause AKI [23, 24]. Neonates in this study were not subjected to any of these treatment strategies prior to the first echocardiography and collection of urine day 3.

In concordance with previous studies we found that U-NGAL decrease with higher gestational age at birth or birth weight and also that U-NAGL is higher in girls than in boys in preterm neonates [8, 9, 25]. Gestational age could therefore partly explain why Tosse et al. report higher U-NGAL levels in neonates with PDA needing intervention compared to neonates with no PDA or a PDA with no need for intervention. Neonates who required intervention in the study by Tosse et al. had a lower gestational age at birth than the neonates with PDA in our study, whereas the non-intervention group and the neonates with no PDA had similar gestational ages as ours. Tosse et al. did not adjust for gestational age in their analysis. When adjusted for gestational age and gender we found no association between PDA and U-NGAL which is in also in agreement with Laundry et al. [9].

AKI is defined as a sudden decline in kidney function resulting in derangements in fluid balance, electrolytes, and waste products. Currently a modified neonatal Kidney Injury: Improving Global Outcomes (KIDGO) classification of AKI accepted at the National Institute of Diabetes and Kidney Disease (NIDDK) workshop 2013 is used [26]. The applicability of these criteria for AKI in neonates is problematic because they include SCr and urinary output. Neonates often have non-oliguric renal failure [27]. SCr is difficult to interpret in preterm neonates due to incomplete nephrogenesis and the potential impact of bilirubin on the measure of SCr. In the first days of life maternal renal function also plays a significant role in the magnitude of newborn SCr due to placental transfer [27]. Previous studies using a plethora of AKI definitions have found an association between AKI or altered renal function and PDA [28, 29]. At the time our study was designed and performed the modified neonatal AKIN classification as proposed by Jetton et Askenazi was used [15]. This definition does not include urinary output. In a cohort of very preterm neonates that all had an echocardiography day 3 to diagnose PDA we found that seven neonates (5%) had AKI within the first week of life. We found that AKI was associated with gestational age, but not PDA.

The renal function in premature neonates is affected by both immaturity with a decreased ability to reabsorb electrolytes and protein and to concentrate urine and by an increased risk of injury during the early postnatal period due to multifactorial morbidity, medication, umbilical artery catheter, and hemodynamic instability [26, 30, 31]. Urinary albumin is inversely related to gestational age, which may reflect tubular immaturity, tubular damage and glomerular dysfunction [12, 30, 32]. We found urine albumin to be associated with PDA and PDA size also when adjusted for gestational age, gender, and sepsis. A recent study found urinary albumin to be increased in preterm neonates treated with indomethacin compared to neonates that did not receive indomethacin. No information on the PDA was given in that study [33]. Urinary albumin has been found to be increased in preterm neonates with asphyxia and respiratory distress and has been suggested as a possible biomarker of AKI in newborns [32, 34] and preterm neonates [35]. In concordance with our findings FENa is known to be very high in preterm neonates and inversely related to gestational age [36]. Several mechanisms may cause this including immature tubular function, low density of sodium transporters in the tubular epithelium, and a physiological response to redistribution of extracellular fluids. After adjusting for gestational age, gender and sepsis we found no association between FENa and PDA.

Due to the difficulties in diagnosing AKI in preterm neonates, studies have investigated different biomarkers in order to allow also for earlier identification of neonates with AKI [26, 37]. An association between AKI and U-NGAL has been described in preterm neonates [8, 9, 38–40]. The definition of AKI differed in these studies, but they all included SCr. We found that U-NGAL was not an early marker of AKI in our population as we found no association between U-NGAL day 3 and AKI. Furthermore U-NGAL day 6 was not associated with AKI. In concordance with our findings Gubhaju et al. found no association between AKI and U-NGAL [30]. Gubhaju et al. recently described an association between U-NGAL and urine total protein, but contrary to us they did not find a positive association between U-NGAL and urine albumin [30]. Increased U-NGAL in extremely preterm neonates may be associated with infection, inflammation or indicate differentiation and growth of renal epithelium caused by immature nephrons stimulating glomerulogenesis [38, 41, 42]. We found U-NGAL to be associated with sepsis in very preterm neonates.

In our analyses we adjusted for gestational age, gender, and sepsis as previous studies found that they may influence U-NGAL levels. We included a total of 146 very preterm neonates in this study. Despite this being the largest cohort evaluating U-NGAL in preterm neonates, a larger cohort would be needed to allow control for more covariates and to facilitate subanalyses by gestational-age. Day-to-day variation has been described in both U-NGAL and SCr. Unfortunately; we could not collect all samples at the exact same time after birth. However, the variation in time of collection was similar in neonates with and without PDA. Renal complications in neonates with PDA may be due to hemodynamic effects of the PDA or to treatment strategies used. We were able to measure U-NGAL (day 3) before Ibuprofen was administered. However, all but one of the six neonates with PDA that were diagnosed with AKI received Ibuprofen in the interval between the two measurements of SCr used to define AKI.

## Conclusion

In this cohort of 146 very preterm neonates we found no association between U-NGAL and PDA day 3 after adjusting for gestational age and gender. We found that PDA day 3 was associated with urine albumin; however PDA was not associated with AKI and FENa when adjusted for gestational age and gender. No association between U-NGAL and AKI was found. U-NGAL day 3 was associated with both urine albumin and FENa. Non-renal disease may constitute important confounders in the interpretation of NGAL and highlight the need for an enhanced understanding of the interactions between cardiovascular function, inflammation, AKI, and the risk of adverse outcome. At the present time U-NGAL is not useful as a diagnostic marker to identity very preterm neonates with a PDA causing hemodynamic changes resulting in renal morbidity within the first week of life.

## Abbreviations

AKI: Acute kidney injury
FENa: Fractional excretion of sodium
GA: Gestational age
PDA: Patent ductus arteriosus
SCr: Serum creatinine
U-NGAL: Urinary neutrophil gelatinase-associated lipocalin
